# Perceptions of Peer Support for Victim-Survivors of Sexual Violence and Abuse: An Exploratory Study With Key Stakeholders

**DOI:** 10.1177/08862605211007931

**Published:** 2021-04-15

**Authors:** Alison Gregory, Emma Johnson, Gene Feder, John Campbell, Judit Konya, Concetta Perôt

**Affiliations:** 1 University of Bristol, UK; 2 University of Exeter Collaboration for Academic Primary Care (APEx), Exeter, UK

**Keywords:** peer support, sexual violence, sexual abuse, sexual assault, qualitative

## Abstract

Experiences of sexual violence, childhood sexual abuse, and sexual assault are common across all societies. These experiences damage physical and mental health, coping ability, and relationships with others. Given the breadth and magnitude of impacts, it is imperative that there are effective, accessible services to support victim-survivors, ease suffering, and empower people to cope, recover and thrive. Service provision for this population in the United Kingdom is complex and has been hit substantially by austerity. Since positive social support can buffer against negative impacts, peer support may be an effective approach. The aim of this exploratory study was to capture the views and perspectives of professional stakeholders concerning service provision for victim-survivors, particularly perceptions of peer support.

In-depth semistructured interviews were conducted in the UK during 2018 with six professional stakeholders, highly experienced in the field of service provision for victim-survivors of sexual violence and abuse. An abductive approach to analysis was used, applying principles from thematic analysis. Our sample comprised four females and two males, and their roles included psychiatrist, general practitioner, service improvement facilitator, and senior positions within victim-survivor organizations.

Interviews highlighted models of peer support for this population, good practice and safety considerations, and a lack of uniformity regarding quality and governance standards across the sector. Findings indicated that current funding models impact negatively on victim-survivor services, and that provision is fragmented and insufficient across statutory and not-for-profit sectors. The influence of the medical model upon service provision was evident, which resulted in apprehension around support delivered in less-usual forms—including peer support. Further research is needed to explore the potential of peer support for victim-survivors of sexual violence and abuse.

## Introduction

Sexual violence and abuse are pervasive in every society (WHO, 2002). In the United Kingdom (UK), 0.5% of children aged under 11 years old, and 5% of 11–17 years old report having experienced sexual abuse by an adult or peer (Radford et al., 2011). Reports of sexual abuse at any point during childhood rise to over 11% (5% of men, and 18% of women) when the question is asked of people aged 18–24 (Radford et al., 2011). These figures are subject to underreporting with at least one in seven not disclosing their experiences (ONS, 2019). Experiences of sexual violence and abuse are not limited to childhood. The Crime Survey for England and Wales estimates that 20% of women and 4% of men have experienced sexual assault since the age of 16, with 560,000 women and 140,000 men sexually assaulted in the past year (ONS, 2018, 2019). Indeed, it is estimated that, in the UK, eleven adults will experience rape, attempted rape or sexual assault by penetration every hour (MOJ, 2013). Experiences vary hugely; some victim-survivors^1^ have suffered one incident of sexual violence and abuse, others have experienced repeated victimization, either by an individual perpetrator over time, or by multiple perpetrators (Classen et al., 2005; Cox, 2016). Some victim-survivors have been sexually assaulted or abused by a stranger, many more have been assaulted or abused by someone they know, often attachment figures in childhood (Cox, 2016; Merrick et al., 2018; Smith et al., 2015).

The experience of sexual violence and abuse damages health and wellbeing. It may affect an individual’s physical and mental health, their ability to cope and seek help, their relationships with other people, and their socioeconomic participation. In addition to injuries, victim-survivors are more likely to have immediate and longer term physical health conditions, including: urinary tract symptoms, sexually transmitted infections, chronic pain, gynecological conditions, coronary heart disease and diabetes (Almuneef, 2021; Anderson et al., 2014; Felitti & Anda, 2010; Lampe et al., 2003; McFarlane et al., 2005). Victim-survivors are also at significantly higher risk of poor mental health, experiencing conditions such as post-traumatic stress disorder (PTSD), depression, anxiety, and struggling with self-harming and suicidal thoughts and behaviors (Chen et al., 2010; Ellsberg et al., 2008; McFarlane et al., 2005; Pico-Alfonso et al., 2006; Wong et al., 2019). Moreover, people who have experienced sexual violence and abuse are more likely to engage in hazardous behaviors including harmful drinking, taking illicit substances, and engaging in risky sexual behaviors (Campbell et al., 2009; Thompson et al., 2017; Wong et al., 2019). Victim-survivors are more likely to develop eating disorders and to experience poorer social relationships (Chen et al., 2010; Wong et al., 2019). Sexual abuse in childhood is a significant risk factor for sexual revictimization in adulthood (Ports et al., 2016). These social, relational and health impacts may be lifelong (Fleming et al., 1999; Mullen et al., 1994).

Given the prevalence of sexual violence and abuse, and the magnitude of harm, it is imperative that there are effective, accessible services to ease suffering, and to empower victim-survivors to cope, recover, and thrive. The National Institute for Health and Care Excellence in the UK recently published a review regarding nonpharmacological interventions for the treatment of PTSD symptoms (NICE, 2018). Based on their detailed review of the available empirical evidence, their treatment recommendations were Trauma-Focused Cognitive Behavioral Therapies and Eye Movement Desensitization and Reprocessing. While these forms of therapeutic support may, of course, be helpful in relieving trauma symptoms, the review highlighted additional points of importance. First, that different subpopulations experiencing trauma symptoms have different prognoses, therefore we cannot assume that what works for one group of people will work for all. Second, while the review shows us what interventions may be helpful with regards to PTSD symptoms, it indicates little about the many and varied additional health, well-being, and relational impacts that people may experience following traumatic events. And third, it indicated the dearth of research about many types of nonpharmacological intervention. For example, the review identified only two papers describing peer support interventions, both excluded for having unusable data (NICE, 2018).

The guidance produced by The National Institute for Health and Care Excellence is primarily applied in healthcare settings. However, the landscape of services in the UK for victim-survivors of sexual violence and abuse is complex, spanning multiple sectors and government organizations, including health, social care, and justice (NHS England, 2018b; Smith et al., 2015). Additionally, the commissioning of UK services, often to third sector organizations, varies by locality, with funding increasingly devolved to a local level in recent years (Green & Skeates, 2018). Compounding these challenges is the devastating impact of austerity: cuts in UK statutory service provision and third-sector income, leading to service closures, restrictions, and extensive waiting lists (Halliday, 2015; Reis, 2018). According to the UK All-Party Parliamentary Group on Sexual Violence, “Sexual violence and abuse support services have been described as at crisis point,” with many organizations existing on a “hand to mouth basis” (Green & Skeates, 2018). Where does this leave victim-survivors? Frequently, they are trying to navigate a landscape in which provision is confusing, shifting, opaque, and disjointed, while struggling with debilitating trauma symptoms, only to find that available resources are scarce, often inaccessible and short-term, and may have waiting times of more than 12 months (Green & Skeates, 2018; NHS England, 2018b; Smith et al., 2015). Some victim-survivors fund their own support with private therapists, but this increases inequality of access and creates a moral hazard: expecting people to pay for essential services to support recovery from crimes committed against them. The UK Ministry of Justice’s recent commitment of an extra £5 million to support services for victim-survivors of sexual violence and abuse (MOJ, 2019) is widely acknowledged to fall short of what is needed; it simply “won’t undo the chronic underfunding” which has existed for decades (EVAW, 2019). In the UK, the COVID-19 pandemic has focused political attention on this short-fall, with the promise of a short-term injection of funding, but confidence is low that increased funding will continue once the crisis has passed.

There is a disparity between level of need, availability of service provision, equity of access to services, and funding. Therefore, it is imperative to consider whether there are forms provision which are supportive, effective, and cost-effective, so that the same limited pot of money can serve a greater number of individuals. Initiatives, such as Social Prescribing (NHS England, 2018a), have been trailed in England and have been shown to be helpful for some people with complex needs affecting their wellbeing, by connecting them to community groups for practical and emotional help, and the therapeutic benefits derived from different activities. One of the possibilities for consideration, and increasingly the subject of research with people experiencing poor mental health, is peer support (Crisanti et al., 2019; O’Connell et al., 2017; Rüsch et al., 2019). Of course, any such use of peer support needs to avoid the burden of care being placed on victim-survivor communities, and to retain the acceptability, accessibility, beneficial gains, continuity, and sustainability of other provisions for victim-survivors so that a range of high-quality support is available. NHS England describes peer support as follows:

…a range of approaches through which people with similar characteristics (such as long-term conditions or health experiences) give or gain support from each other to achieve a range of health and wellbeing outcomes. These include building people’s knowledge, skills and confidence to manage their condition and improving quality of life and social functioning. (NHS England, 2016)

These approaches include support, which is entirely peer-developed, peer-instigated, and peer-led; support which is primarily developed, instigated, and led by professionals; and a whole range of permutations in between. It can describe support which is group-based or one-to-one, and it can be delivered in-person, over the phone or via a range of virtual media. Models of peer support may involve elements of listening, psychoeducation, tutoring, mentoring, activism, and advocacy, and are underpinned by the benefits of nuanced understanding, and greater empathy and respect that peer supporters are perceived as offering (Mental Health Foundation, 2013).

Research with victim-survivors has demonstrated that positive social support, in all its forms, acts as an important buffer against negative impacts on health, well-being, and quality of life, and may increase people’s resilience (Cénat et al., 2019; Fuller-Thomson et al., 2020; Machisa et al., 2018). Thus, it is perhaps not surprising that models of peer support (both structured and unstructured), which center on the importance of social relationships, might prove beneficial. Additionally, there is evidence that victim-survivors prefer to be supported by people who have themselves had related experiences, because feeling listened to, believed, respected, and understood is more likely with peers (Gray et al., 1997; Robotham et al., 2019). The UK Department of Health has funded research with victim-survivors which highlighted this desire for peer support to be readily available (Scott et al., 2015). In a recent systematic review, focusing on peer group support for victim-survivors of sexual violence and abuse, we reported that group participants experienced several benefits from peer support. These included: experiencing empathic witnessing resulting in feeling genuinely understood; finding permission to acknowledge and accept the reality of experiences; increased social connectedness; impetus towards healing from witnessing others’ progress; and opportunities to discover resources and coping strategies (Konya et al., 2020).

Our review demonstrated that the evidence base for peer support for victim-survivors, while promising, is also limited; only eight articles were found, most were pre-2000s, and paper quality was low. Victim-survivors themselves identify support from peers as a research priority; appreciating that this type of support can be helpful, but also the limited evidence base and limited understanding about how to “create opportunities for peer support” (Robotham et al., 2019). Research on this topic is thus both necessary and timely and should include an understanding of the current service landscape from the perspectives of people who design, deliver, provide, signpost to, and commission services in the sector. The aim of the study reported here was exploratory: to capture the views and perspectives of professional stakeholders concerning service provision in the UK for people who have experienced sexual violence and abuse. In particular, we wanted to gain an understanding of how forms of peer support were perceived, in order to provide a foundation for the development of future services.

## Methods

A qualitative approach was untaken because it captures people’s experiences and beliefs in depth, in order to provide fundamental comprehension of that which is complex (Reid, 1996). It is an approach that has been widely used in health services research, including to gain an understand of the beliefs, attitudes, and perceptions of professionals (Pope & Mays, 1995). In qualitative research, fewer participants are included, but are chosen deliberately and carefully as people who can provide key information about a phenomenon (Reid, 1996).

### Participant Recruitment

To understand and explore the perceptions of professional stakeholders, we designed an in-depth qualitative interview study. We defined professional stakeholders as people working in the UK in a substantial professional role related to service provision for victim-survivors of sexual violence and abuse. After obtaining ethical approval for the study, from the Faculty of Health Sciences Research Ethics Committee at the University of Bristol, we sought to identify potential key informants. Discussion within the research team highlighted individuals likely to be eligible and interested in participation, and professional group networks through which further individuals might be contacted. The focus of our recruitment was on relevant practitioners, service providers and service commissioners.

Employing a purposeful sampling strategy, a member of the research team (EJ) contacted potential participants via email inviting them to take part in the study. Six expressions of interest were received. These individuals were subsequently emailed a participant information sheet and study consent form and were provided with the opportunity for any questions they had to be addressed. From these initial expressions of interest, all six individuals agreed to take part. The material collected was considerable and, since the nature of the research was very focused, the data were both rich and detailed. After six interviews, a degree of consensus in the data was apparent, and thus the collected data was deemed sufficient to satisfy the aims of this small in-depth exploration.

Our sample comprised four females and two males, age range 44–69 years. Participants, none of whom were known to the interviewer, identified as either White British or White African. At the point of recruitment, participants were working for the UK’s National Health Service (NHS), third sector charitable organizations, or both. They all worked in England, and their roles included psychiatrist, general practitioner (GP), service improvement facilitator, and senior positions within specialist organizations for victim-survivors. The professionals within specialist organizations had considerable experience of working in the area of sexual violence and abuse (between 10 and 25 years) and held a variety of relevant professional qualifications. Some participants indicated that they had had personal related experiences. Although participants had been identified through the professional networks of the wider team, the researcher collecting the data (EJ) had not met any of the participants prior to interview, and only anonymized transcripts were used for analysis.

### Data Collection

In-depth semistructured interviews were conducted by the second author (EJ), either over the telephone (*n* = 5) or in a private office in university premises (*n* = 1), according to participant preference. Interviews were audio-recorded, and participants provided written consent for participation immediately prior to interview. The duration of the interviews was between 40 and 62 minutes. Interviews followed a topic guide designed to elicit participants’ knowledge, perspectives, and views about peer support services for victim-survivors of sexual violence and abuse. Questions explored both personal awareness and viewpoints, and participants’ thoughts about how victim-survivors might perceive this type of support. Additional probing was used to facilitate elaboration and to achieve depth of discussion. Prior to interview, participants had been emailed a one-page summary of a current example of a peer support service: a peer-led group program for adult victim-survivors of sexual violence and abuse being run by a small charity. During the second half of the interview, the summary was used to prompt and encourage further reflection and discussion about the perceived pros and cons of different components or options for peer support.

### Data Analysis

Audio-recordings from interviews were transcribed verbatim and transcripts were cleaned and anonymized. An abductive approach to analysis was employed: initially developing and applying a loose framework to manage data, and subsequently using principles from inductive thematic analysis (Braun & Clarke, 2006) to develop, expand, and hone our analysis.

In the first phase of the analysis process one member of the research team (EJ) imported the transcripts into NVivo 11 software and generated initial descriptive codes which were grounded in the whole data set. In parallel, two team members (AG & CP) each independently coded half of the interviews. The analysis team (EJ, AG, & CP) subsequently met on a number of occasions to discuss the generated codes, and to decide how to refine and organize them. This was an interactive, iterative, and creative process to organize and clarify thinking, identify patterns, overlap, and connections between codes, and ultimately to develop a thematic coding framework.

The second phase of the process involved the analysis team returning to the original data to apply the enhanced thematic coding framework to each transcript. All relevant data pertaining to individual codes were identified, extracted, and organized using a framework in Microsoft Excel. This enabled us to look both across and within each participant and theme. The team met regularly to reach consensus regarding the collapsing and clustering of the most pertinent codes and to generate a final list of themes which were subsequently defined and transformed into a narrative account.

## Findings

Participants’ perceptions and knowledge of peer support services and opportunities for victim-survivors of sexual violence and abuse were explored. In order to introduce the themes generated in the analysis, it is important to understand the wider context of stakeholders’ narratives. This contextual summary includes detail about participants’ understandings of peer support, and their feelings about the appropriateness of this type of support.

It would be remiss not to begin by mentioning the strength of views expressed by participants when presented with an example of an existing peer support service, as part of the interviews. Using this example did, however, prompt a great deal of discussion about the variety of approaches, models and setups that peer support can take. For example, participants talked about peer support taking place face-to-face, in community or specialist service settings, and also remotely through online forums. They discussed formal and informal approaches, including those facilitated or cofacilitated by trained professionals (such as clinical psychologists). There was mention of peer support offered for specific cohorts of victim-survivors (such as older people and those with learning impairments) and based around social and creative activities (for example, walking and crafts). There was a strong sense from the people we spoke to that peer support was certainly not appropriate for everyone. Although peer support was seen as having potential value or benefit for some individuals, at particular stages of their journey postvictimization, participants were keen to stress the importance of peer support being part of a range of services and provision, “to suit different people with different needs. ” In particular, gender, culture, ethnicity, learning disabilities, employment, and caring responsibilities were factors mentioned by participants as having potential bearing on whether a particular service or format was appropriate and accessible. Participants additionally discussed the timing of access and “readiness” for engagement with peer support services, describing more and less appropriate moments within an individual’s trajectory towards recovery and healing. In this way, peer support was viewed as one step as part of a continuum of provision, and as potentially harmful or damaging if a victim-survivor was not ready to successfully manage peer-to-peer relationships, or had not first undertaken individual work with a professional.

These insights into stakeholders’ views about the variety and suitability of peer support provide context for the following themes generated during analysis: medicalization, power, and choice; perceived function and impact of peer support; good practice and safety considerations; the insufficiency and fragmentation of services; and the impact of current funding models.

### Medicalization, Power, and Choice

As we spoke with participants about their impressions of peer support services, evident in their narratives were the ways in which the medical model has influenced and shaped services for victim-survivors. This influence appears to have impacted on decision-making power, and on how choice is offered or restricted.

With regards to the medicalization of services, professionals were described as the “experts,” with their knowledge, professional experience, recognized training and qualifications repeatedly described as necessary for service provision:

Everybody here has got just heaps and heaps of trauma training, and experience, and skills…our workers are really skilled and give people stuff that really helps them move on. (P2)

Participants routinely talked about the empowerment of victim-survivors, however, there appeared to a big leap of faith required from empowering victim-survivors more generally, to empowering them specifically to undertake service-delivery roles with their peers:

…they might not have experience or training in actually how to relate or how to provide a counselling, therapeutic relationship with somebody. (P1)

The facilitator will always be a paid worker because then we have control and authority over them, and we take full responsibility. (P2)

Even where services *were* offering peer-to-peer support, participants indicated their desire for a professional to “to keep an eye on” what was happening, fearing that safety might be being compromised. Safety was mentioned repeatedly throughout the interviews as the overriding concern for victim-survivor organizations, though a few participants questioned the appropriateness of this:

I think often safety can be a bit of an illusion because the world isn’t per se a safe place. What we want is people to be able to manage themselves in a relatively unsafe world, I guess. (P6)

Further features of the medical model include its commitment to evaluation, rigor, routine monitoring, and evidence-based practice. Participants felt strongly that services for victim-survivors should adhere to these values, though also indicated that there were important holistic changes experienced by people which are subtle, nuanced and rarely captured, such as: feeling understood, restoration of hope, a sense of connection, experiencing joy, regaining life structure, and doing “normal things”:

…all those things, they’re not just about people sorting out their psychological difficulties but they’re about recognising the difficulties that are faced by, in this case, sexual abuse, perhaps impacts and ripples in so many aspects of their lives. If services don’t recognise that then they’re not doing people a service. (P6)

One of the concerns expressed about peer support was that services might not incorporate evidence-based practices; although interestingly, much of the practice cited by participants was practice-based evidence (where real-world practice was being evaluated *in situ*) rather than necessarily evidence-based practice.

In addition to modes of practice, the language used by participants was frequently medicalized, for example: “clinical need,” “treatment,” “symptoms,” “diagnosis,” “models,” “interventions,” “screening,” and “appraisal.” Moreover, an impetus for change and movement by victim-survivors was conveyed by terms such as “move on,” “progress,” “process,” and “pace.” Herein, there was also a degree of confusion around whether peer support services were delivering therapy, or support which was therapeutic. This confusion left participants feeling that, on the one hand, peer support was offering something potentially “supportive” and “holding,” and on the other hand, that support from peers might be “habit-forming,” encouraging “wallowing,” and even “helplessness”:

...I think it makes people helpless in a way…. You can’t keep rehashing the same stuff. If you're doing proper work I think, then you make those changes so that the emotions attached to them are no longer.... The memory is still there, but the emotions are not as viable as they were before. (P4)

Regarding decision-making power, participants gave some examples of decisions which had been shared or negotiated with victim-survivors:

I think it’s about just keeping an open view and consulting with the survivor through the process…. I think one of the things we want to avoid always is having limiting criteria for people to access services. (P3)

Examples were also given where practitioners made gatekeeping decisions and judgements about whether individuals were “a good candidate for this sort of work” and, where people had not been made aware of, or offered, the full range of available options.

In terms of input within the *development* of services, most participants talked about the valuable insight and steer that victim-survivors could give, acknowledging that they are “well placed to have a sense of how it affects them and might affect other people.” However, participants also indicated instances where decisions about provision had ultimately remained in the hands of the professionals, a common feature in the medical model.

### Perceived Function and Impact of Peer Support

The second theme relates to how peer support was understood in terms of both function and impact. Participants’ reflections on peer support were dominated by narratives about group-based provision. We suspect that this was for two reasons; the first is that the most widely publicized peer support services are delivered in group settings (e.g., Alcoholics Anonymous), and the second is that participants had been presented by the interviewer with an example which was group-based. Regarding the group-based form of delivery, participants had mixed feelings. On the one hand, they highlighted possible positive impacts for psychological and interpersonal wellbeing, describing groups as potentially supportive environments where people would have opportunities to make friends, to share their experiences, and “be with others who know” and understand:

I think there’s something very, very powerful around just being in a space. You don’t have to talk about the details of the abuse but just knowing that you’re with people who have experienced something similar to you is very powerful. (P3)

Peers were seen to be well placed for having unique insight into one another’s experience and for assisting each other to “move through and integrate that experience into their lives in really helpful ways.” Positives around reduction of self-blame and internalizing behaviors were also mentioned:

I mean, actually, if you’re in there with people who can very clearly say, “No, that clearly wasn’t your fault,” then that might be actually very helpful to hear that from other people who’ve been in similar situations. (P1)

Spending time with other survivors was viewed by participants as a way to reduce isolation, a common issue for victim-survivors:

It’s the fact that it has happened to the person beside me…I think it raises an awareness that you’re not alone, I suppose. Maybe it takes away the isolation. (P4)

Participants also suggested that peers might find it helpful to share information about organizations and resources, and about coping strategies for managing trauma and distress symptoms. One participant additionally reflected on the potential of positive role modeling that leaders might offer to fellow peers, particularly around agency and hope:

…that person who’s leading may well have come through an awful time. They may be able to share, give them hope. They may have a lot more experience in terms of an awareness of what other agencies are out there, and signposting kind of thing. (P5)

Conversely, participants remained cautious about some of the risks they felt were possible with peer support delivered in group settings. Beyond the idea that disclosure in a group could leave a person feeling exposed, were more specific concerns about friendships between peers having negative impacts:

They can make friends to begin with and then it all goes horribly wrong. Then, some people are pushed back to a lower level than they were beforehand. It could all seem quite nice at the time…. (P4)

Moreover, a shared worry among participants was that hearing others’ experiences could have a negative impact; with peers potentially being triggered by hearing other people’s disclosures or feeling a sense of burden:

Some people find it traumatising listening to other people’s stories as well, and then that retriggers their own difficulties again. (P5)

### Good Practice and Safety Considerations

Related to the risks participants perceived regarding peer support, particularly support delivered in group contexts, people highlighted considerations for ensuring that provision of services is both high quality and *safe*. These reflections related principally to peer facilitator expertise, group member suitability, and organizational features and processes.

With regards to facilitators, who were themselves peers, the concerns centered on whether individuals were suitably qualified and sufficiently supported to undertake this role. Participants were emphatic that suitable qualifications and training, particularly in trauma-focused support, were a minimum requirement, and only small emphasis was given to the expertise that comes from lived experience of trauma and the daily management of its impact. People expressed concerns that peer facilitators might lead support groups without sufficient skills and expertise:

Because they’re not professionals, they may not be trained in how to sort of present, or they may give their own opinions that may be disturbing to others. (P5)

…if someone went into something like a flashback or had an adverse reaction in a group, how that would be managed within a group and, I guess, who would manage that and whether people would be qualified to manage that. (P3)

Participants recognized that professionals might also lack skills and expertise around managing groups, but felt that there was an increased likelihood if the person leading the group was a peer rather than someone with substantial training about the management of trauma and distress. Additionally, since supervision and monitoring were seen as vital by participants for ensuring self-care and close alignment with the evidence base, concern was expressed about services which may not incorporate these tenets, particularly peer support services:

…I would worry about people providing treatment for PTSD or any other mental health outcomes related to having [experienced] sexual assault, without close monitoring and supervision, and without following some kind of evidence base. (P1)

One participant, who provided an example of a peer support program successfully run by her service, described how recognition of this supervision need has led them to put in place a safety net for their peer mentors:

…a different person leads it on a weekly basis, and they decide within the group who that is…that person will debrief with the organisation at the end of the group. So, they’ll debrief with a specific support worker and just let them know how the group went, who attended, that sort of thing. (P3)

Considering group member suitability, participants shared examples of screening and assessment processes for people who entered their services, in order to minimize risks, to assess if the format of the provision was appropriate, and to afford protection to everyone. Participants expressed an uneasiness about peer support services in this regard, feeling that these services might forego suitability considerations:

We want to ensure that people are ready to be able to manage peer relationships and things like that before they go into a peer led setting because it can be quite damaging… There are definitely some people who will come forward who wouldn’t be a good candidate for this sort of work. That needs to be handled carefully and sensitively. (P3)

It’s difficult, isn’t it, because if you’re going to say, “free to attend for everybody,” then it’s difficult to start sitting them down and making an assessment on it, isn’t it?…but then you could be putting your group leaders and your peer leaders at harm by some of the people that turn up. (P4)

Linked with this were concerns about peer support services offering insufficient boundaries to people who, as a result of the sexual violence and abuse perpetrated against them, may struggle with maintaining these. Participants felt it would be hard to uphold firm boundaries between peers both within and outside of the support context:

People have had a lifetime of being judged and their boundaries being walked over, so when you get into these groups, when someone walks over your boundaries and you’re walking over theirs, then it can be quite volatile for them, in my opinion. (P4)

…you might end up with two people developing a relationship outside of the group, being disruptive…. I think they’re littered with pitfalls, peer support groups, and I think they need to be managed and governed quite carefully and quite well, in terms of the safety aspect. (P3)

Participants also spoke fervently about good practice and safety considerations at an organizational level. They described the requirement for services to be transparent, accountable, well-governed, and ethical; and for there to be regular assessment, monitoring, and evaluation of the quality of services provided. People described some frustration about the lack of uniform quality assurance processes and standards within the sector, and were concerned that peer support services, in particular, might fall short:

…it doesn’t matter whether they’re peer led or professional led, the same issues apply, I think about making sure that what you’re providing is clearly set out, clearly adhered to, monitored, evaluated. (P1)

Well, one that we found in other contexts is because [peer support services] tend to be done very cheaply by people who meet on a volunteer basis, it can be very hard to measure their impact and their outcomes, when actually I don’t think there’s any service that you wouldn’t want to measure. (P6)

### The Insufficiency and Fragmentation of Services

Beyond the concerns about consistency in terms of quality and standards, a strong theme emerged around the insufficiency of provision, statutory or otherwise, for people affected by sexual violence and abuse. Participants’ narratives included the issue of fragmentation of services, inequality of access related to geographical spread, waiting list length, and quality of interventions that were deemed too brief:

What they aren’t able to provide is longer term, more focused help for difficult problems…they usually offer around six sessions maximum…they might be able to do something that’s just getting over the current problem or current issues, but in terms of really tackling the underlying PTSD issues, then that’s unlikely to be enough. (P1)

Information exchange about existing services (including peer support services) and formal referral pathways was often unclear and, counterintuitively, the more complex the trauma experienced, the longer the likely wait for any service:

Well, maybe three or four months [wait]. If it’s complex PTSD which the majority of them are, then it’s 18 months and has been an 18-month-long waiting list since I started in the service, about 15 years ago. It hasn’t changed. (P1)

One participant described their service’s attempts to ameliorate the problem of waiting lists:

We recognise that survivors who refer into our service are going to feel really anxious and making contact with them as early in the process as possible and then keeping up that contact throughout the time that they’re waiting has proved invaluable…. Anyone on the waiting list will receive a weekly or two weekly phone call. They determine that frequency. Just to check in with them, see how they’re doing. Things can change. You know, someone can move from being quite well supported and feeling in an okay place to being not so great quite quickly. (P3)

In addition to describing services for victim-survivors as inadequate, participants also noted the fragmentated, often siloed, approach between the different sectors working with victim-survivors, particularly criminal justice, health, and third sector specialist organizations. Participants felt that what was required was not just signposting between services and mutual promotion, but also the development of referral pathways, and cross-sector training:

But, for me, I’ve done those jobs for 20 years, and there was definitely siloed working, and in my mindset, I didn’t think across strands... actually, we should be looking more at, holistically, people’s life experience and why they’re coming to us. (P5)

…I’m wanting to make sure that it provides connection with current domestic and sexual abuse services…. We need to make sure that mental health provision covers these issues appropriately or connects with organisations and services. (P6)

In exploring systemic issues regarding lack of capacity, this commissioner participant, highlighted the potential advantages of peer groups to help tackle this:

A lot of our services in mental health are hugely expensive. You’re talking about consultants, over £100,000 a year just for one post, one person. It’s crazy money, isn’t it? Whereas peer-led groups and support structures, mentoring support, things like that can be incredibly supportive of people and changing of their lives with a minimum of financial input. I have more of a principle-based view of their efficacy, but we can’t ignore the money thing. (P6)

Another participant, however, expressed more cynicism about using peer support, particularly groups, to bolster provision:

I think, most of the time, they’re about money. So, it’s, “Hey, it’s quicker to do it in a group.” (P4)

What participants seemed to be underlining was the importance of offering peer support on its own merits rather than viewing it as a way of covering up the cracks in service provision.

### The Impact of Current Funding Models

Intrinsically linked with the reality of insufficient provision, participants’ narratives highlighted how current models of funding within the sector have led to challenges for victim-survivor organizations. In particular, people mentioned inconsistent provision across the country and the shifting landscape, which they felt was about individuals and politics rather than an evidence-base or fundamental recognition of need:

…funding is dependent upon personality and politics. If something is high up politically, then funding will be available. If personalities change or politicians change, then that shifts…. I think it is about political will. (P2)

This financial insecurity impacted the day-to-day delivery by organizations; participants emphasized the unacceptable length of waiting lists (with some describing 18 months+ as standard), and the constant pressure to demonstrate “change” in order to secure future funds:

It’s great that people have started to sort of realise there are agencies out there that can support them, but they then become overwhelmed because there’s a time lag where the services aren’t there, you know, unless funding increases. (P5)

Participants also discussed how the lack of financial continuity was a deterrent to infrastructure development. This sometimes meant that organizations relying heavily on volunteers, found themselves in a catch-22 situation; without enough consistent workers to measure and demonstrate value, and consequently appearing undeserving of the funding which would enable necessary infrastructure development:

…in terms of those services often not having an infrastructure and paid workers and time other than the input, they often end up not having the capacity or the resources to measure themselves very well…. (P6)

The *source* of the funding was also seen as important, with public funding viewed both positively and negatively. On one hand, public funding might be more dependable, and invested in evidence-based, accountable services. On the other hand, participants expressed frustration at the rigidity encountered with this funding, meaning that organizations did not have options to sufficiently tailor their services.

In addition to challenges created by current funding models *within* organizations, it was apparent that competitive tendering added to any tensions *between* organizations. This could result in defensive positions, as organizations sought to protect themselves. Sometimes these tensions were more explicit, apparent in the strength of participant’s views and emotions:

*We* are not just like [our neighbour organisation] and people like that. They write their own reports…about what went on. Then, if you speak to the person, it could be something completely different. *We* ask our clients to assess us. (P4)

On other occasions, these tensions were more hidden within people’s reflections, indicated by their choice of words. For example, people described their own services as “good,” “great,” “evidence-based” and “robust,” and other services as “bad,” “gimmicky,” “concerning,” and “cliquey.” One participant while reflecting on this, described the virtue and superiority that professionals tend to attribute to their own services, or to those with a similar ethos:

…you have to be as open to measuring what doesn’t work, including any damage you’re doing. That’s a difficulty. I can think of many services who think they have a kind of moral high ground that what they’re doing is brilliant and is amazing and often, people talk up, professionals talk up services. (P6)

Having said this, participants knew varying amounts about other services available locally and nationally. Some had very partial knowledge, others knew more and were keen to signpost victim-survivors, particularly if an alternative service was considered closely aligned in values, and offered something different than they were able to:

… I’m just a great advocate in there being a broad range of services. Just because there is a consistency between what people have experienced doesn’t necessarily mean that they need the same response. I think that people should be afforded variety in terms of what they can access. (P3)

Moreover, despite occasional disparaging remarks at a service-to-service level, there was a general sense in which people were keen to move away from silo working, in order to offer victim-survivors more variety, more tailoring, and the possibility of shorter waiting times:

I’m very much in support of more and more services we can provide and the range of different types of services to suit different people and different needs. (P1)

## Discussion

From the interviews with professional stakeholders, it was clear that there were strong views, both positive and negative, connected with the idea of peer support for victim-survivors of sexual violence and abuse. In particular, participants’ concerns centered on the suitability of peer support for every individual, and many questioned the safety of support without professional oversight. Participants were able to easily envisage and verbalize the potential benefits of peer support. They were, however, cautious about the possibility of victim-survivors being triggered or feeling a sense of exposure. While rarely stated explicitly, the narratives indicated the pervasive influence of the medical model in gatekeeping, shaping and guiding services, and the discomfort, confusion and suspicion engendered by forms of support (including peer support) which have a different underpinning. Safety considerations were mentioned throughout the interviews as paramount and within these discussions were ideas about quality and uniform standards of provision, appropriate qualification and skillset of staff/volunteers, and the maintenance of appropriate boundaries. Talking about the sector as a whole, participants described service provision as fragmented, with inequality of access, frequent silo-working, and insufficient resource to provide timely, appropriate care. From the narratives, it was also clear that current funding models inhibit innovation and development, and create a degree of division between different (potentially competing) organizations. Additionally, there was some suspicion around the interest in peer support for victim-survivors, with participants fearing that it may be seen as an inexpensive way to bolster or replace existing support without due consideration for safety and suitability.

The development of specialist “professional” services in the context of mental health or sexual violence services is not straightforward. For example, Stark describes how the professionalization within domestic violence and abuse services, while welcome on many levels, has come at a cost to the fundamental tenets of the movement from which it emerged (Stark, 2007). One of the criticisms of current specialist provision is that in the pursuit of stable funding, hierarchical organizational structures, and the employment of supporters as staff rather than people with lived experience, some of the anger, passion, and momentum of the early movement has been lost. Similarly, in the field of mental health, tensions are described regarding the professionalization of those offering peer support; that the process of becoming a “professional” though training, may interfere with the primary advantages inherent in being a peer (Walker & Bryant, 2013). In particular, it may devalue the unique perspective of peers and their perhaps superior knowledge base (Crossley, 2004; Walker & Bryant, 2013), and there is a risk that “peer support might be in danger of losing its essence and soul; its authenticity” (Stamou, 2014; Stratford et al., 2019).

These debates are important when considering the role and potential of peer support for victim-survivors of violence and abuse; authentic peer support could not only provide a different type and format of service for people seeking support, which Stratford describes as a “breath of fresh air”, but also a return to grassroots social and political empowerment (Stratford et al., 2019). Rather than the empowerment of people *by* professionals, peer support identifies people as the “central agent of their own recovery” (Stratford et al., 2019).

It is additionally noteworthy, that participants made firm distinctions between individuals as professionals and individuals as victim-survivors. This is interesting because we know that there is significant overlap between professional and personal experience for people working within helping professions. For example, Pope and Feldman-Summers found that around 70% of women and 33% of men who were clinical or counselling psychologists had experienced some form of physical or sexual abuse (Pope & Feldman-Summers, 1992). To embrace this, rather than ignore it, creates opportunities for investment in people with relevant lived experience (for example to hone leadership skills) in order to capitalize on people’s potential, particularly in relation to providing support to fellow victim-survivors. In terms of efficacy, reviews in the field of mental health, have found that “peer staff functioned at least as well in these roles as non-peer staff,” and furthermore, that “superior outcomes” regarding the engagement of hard-to-reach populations were achieved (Davidson et al., 2012). A metanalysis by Walker and colleagues, identified that rapport building was more successful by peer supporters than by nonpeers, due to the lack of “professional distance” to overcome, and the enhanced empathy resulting from similarity of experiences (Walker & Bryant, 2013). For the people providing the peer support, they found that this could be an empowering experience, increasing confidence, self-esteem, social networks, a sense of community, and potentially leading to future employment. However, when the peer support was embedded within existing services, there were challenges, with nonpeer staff behaving with paternalism, and continuing to treat peers as users of a service rather than as integrated team members.

While there is little research about peer services for victim-survivors of sexual violence and abuse (Konya et al., 2020), much related research is underway within the mental health field. A recent systematic review, for example, describes the plethora of current research, and indicates disparities between different forms of peer support in terms of outcome, and variations in effectiveness dependent on the particular outcomes measured (White et al., 2020).

Recent NHS initiatives, such as social prescribing (NHS England, 2018a), and an increasing adoption of peer support within mental health services, organizations and charities, and within mental health policy, appear to indicate a shift towards greater acceptance (Scott et al., 2011; Stratford et al., 2019; Watson, 2019). Likewise, in the public domain, conversations on social media platforms, such as Twitter, appear to indicate discourses where peer support is viewed as having potential for people experiencing periods of poor mental health (for examples search: #peersupport AND #mental health). Indeed, peer support for victim-survivors of sexual abuse operates itself on Twitter (#CSAQT), and there are parallel groups on Facebook and Instagram. Compelling research findings are also emerging regarding peer support within the field of mental health; peer support is increasingly being seen as part of the “broader recovery agenda” with emphasis on “user-centred outcomes such as social inclusion and empowerment” (Moran et al., 2020).

This divergence from the medical model in terms of outcomes was also evident in our study, with participants describing the potential for less traditional, more holistic outcomes from peer support, particularly around hope and feeling understood (Carey, 1999; McCormack & Katalinic, 2016). Within the wider peer support literature, mechanisms are suggested, in terms of how hope is instilled and fostered within peer support, which relate to the building of connections, the “normalization” of emotions, particularly those of anger, shame and guilt, (Watson, 2019) and the inspiration drawn from witnessing a fellow victim-survivor becoming “the hero of one’s own life journey” (Davidson et al., 2012). The literature additionally describes the importance of peer support for being deeply understood, particularly since these experiences may be in stark contrast to other life-experiences and a “history of feeling misunderstood” (Stratford et al., 2019). This peer-to-peer understanding, has an inherent reciprocity, with a strong sense of togetherness, as peers share their life-learning and journey together (Watson, 2019). Stratford and colleagues describe how peer support necessarily differs from more traditional hierarchical support, with peers relating as equals and “whole human beings who share…a common sense of humanity.” This sense of bringing your whole self to the relationship links to the emotional attachment, caring and fondness which peers may develop, feelings which Watson describes as *taboo* within professional relationships, but as positive qualities if a peer is to walk empathically, congruently alongside (Stratford et al., 2019; Watson, 2019). Findings from Watson’s review, however, do not solely focus on the positive outcomes of peer support. They also highlight that each element which makes peer support so unique and so valuable, can equally have negative consequences (Watson, 2019).

Related to the potential for negative outcomes, participants in our study expressed areas of concern regarding peer support. Many of these accorded with professionals’ apprehensions reported in the mental health literature; in particular, perceptions that peers may be too “fragile” to undertake the work, and that providing peer support may cause people to re-experience symptoms. Davidson and colleagues make compelling arguments which counteract these concerns, focusing on people’s “persistence and resilience” in dealing with their struggles, as opposed to their potential fragility, and reframing any experiencing of symptoms as an opportunity to role model the determination necessary to get through a difficult period (Davidson et al., 2012). Moreover, the Survivors’ Voices charter highlights that “distress does not automatically lead to damage” (Perôt & Chevous, 2018). Safety considerations and boundaries were additionally discussed by our participants and seen as paramount; underpinning these discussions was the fundamental idea of “risk” and “risk management.” There appeared to be a degree of assumption that clinical and/or professionally delivered services would automatically be “more safe,” and questions were raised about how to minimize risk within the context of peer-provided support. These mirror some of the discussions within the sector as a whole regarding the desperate need for safety standards, quality assurance, and a robust evidence base for *all* services. However, with regards to peer support, there is an additional consideration which presents a dilemma. The ethos and underlying philosophy of peer support is distinct from usual mainstream services, which tend to be based on a medical model. Scott and colleagues describe the commonality between all peer support philosophies, stating that “all are grounded in a recovery philosophy and thus emphasise self-determination, mutuality and the honouring of their peers” (Scott et al., 2011). In their paper on risks within peer support services, they describe peer supporters grappling with the wider mainstream risk discourses as they seek to reformulate risk through a different philosophical lens. By drawing on the “dignity of risk” tenet from the disability field, peers seek to provide support for people which enables them to take risks as part of remaking their lives. This shifts from a position of “risk consciousness” where all possible risks are to be managed and reduced, to an emphasis on having the difficult and honest conversations with peers and trusting them to manage themselves. Within this position, the existential anxiety of risk (particularly when it relates to self-harm and suicide) is tolerated, and the peer is no longer “objectified as a symptomatic person in need of management,” but seen as “a person undergoing a personal crisis which was also a learning opportunity” (Scott et al., 2011). Similar, perhaps, to the thoughts and feelings our participants shared, Scott and colleagues describe how the services in their study struggled to appear credible in the eyes of clinicians, mainstream services, and funders because of this dissonance in philosophical stance. Once the peer services were embedded, however, perceptions had altered (Scott et al., 2011).

Our participants were not alone when it came to concern about peer support being seen as an inexpensive way to bolster or replace existing support. Again, from the field of mental health, the literature indicates staff members’ fears that peer supporters would be taken on by their organization as cheap labor, potentially leading to reduced job security (Stratford et al., 2019; Walker & Bryant, 2013). In this literature, worry was also expressed that policy makers might view peer support as a way to cut costs. However, in response to these concerns, there was also a feeling that peer support is increasingly being seen “as a complement, rather than as an alternative, to existing services” (Stratford et al., 2019).

Some of what our study participants described relates to the sector as a whole, and yet the sector itself remains somewhat undefined; we have little sense of what the *shape* of the sector is. Thus, many of the findings speak not only to forms of peer support, but also to the whole spectrum of service provision for victim-survivors. Research is needed across the sector to map, define, and outline what currently exists, and to evaluate the developmental needs both nationally and within front line provision. Additionally, due to the lack of research about peer support for victim-survivors of sexual violence and abuse, much of the literature included within this discussion has come from other fields, particularly mental health. Careful consideration and appropriation of the learning about peer support into the field of sexual violence and abuse, may be an appropriate next step. Evidence is needed to explore the potential, create opportunities, and to foster the development of peer support for victim-survivors of sexual violence and abuse, particularly research which has consultation with victim-survivors at its heart (Robotham et al., 2019).

### Strengths and Limitations

A key strength of this study is the originality of perspective: by speaking to those working in professional roles within this sector in the UK, we have garnered views rarely sought. Capturing the views of professional stakeholders is an important first step on the road towards establishing the potential of peer support for victim-survivors. Since this was an exploratory study, the primary limitation is that the number of participants was small; we can thus only claim to have captured a subset of stakeholder views. However, we were able to delve down and capture people’s views in depth. The second, related limitation, is the relative homogeneity of participants regarding age and ethnicity, despite efforts to recruit a more diverse sample. In common with other qualitative studies, a degree of caution should be exercised in considering the transferability of our findings beyond the context in which the data were collected. The reported findings relate specifically to the UK context, so it is possible that had the research been conducted in other countries, particularly where the structure and provision of healthcare and victim-survivor services contrast, that findings may have differed.

## Conclusion

Peer support may fill an important gap in the provision of support for victim-survivors of sexual violence and abuse. To understand why peer support, while established in the mental health sector, has not been wholeheartedly embraced by organizations specifically serving victim-survivors, it is vital that we seek the perspectives and understandings of relevant professionals. Dialogue with stakeholders demonstrated the influence of the medical model in the shaping of services, and highlights the context of such services, in terms of insufficiency and fragmentation, as intrinsically linked with current funding models. While professionals view elements of peer support as valuable, they remain apprehensive about support delivered in unfamiliar forms. The underpinning assumptions for this caution are contestable, since they equate professional qualification and delivery with safety, and frame the issue of safety as solely concerning risk and risk management. Since victim-survivors themselves are keen to explore the potential of peer support, and indeed, instigate such provision at grassroots level, further research is needed to equip people with a robust evidence base.

## Note

1. While we recognise the limitations of the shorthand term ‘victim-survivor’, we use this term to simultaneously recognise that the person has been subject to a criminal offence and to acknowledge the possibility of recovery and healing. We recognise and value that people self-define differently from one another and at different time points.

## Authors Biographies

**Alison Gregory**, PhD, is a research fellow in the Centre for Academic Primary Care at the University of Bristol. Alison has a specialist interest in the resultant trauma from domestic and sexual violence and abuse. Alison is a mixed methods researcher with a strong commitment to knowledge exchange and research impact and works closely with related expert organizations.

**Emma Johnson**, PhD, is a senior research associate in the NIHR Health Protection Research Unit in Behavioural Science and Evaluation at the University of Bristol. Emma has a background in health psychology and is an experienced qualitative researcher.

**Gene Feder**, MD, FRCGP, is a professor of primary health care in the Centre for Academic Primary Care at the University of Bristol. Gene’s research focuses on health care responses to domestic violence globally. He chaired the National Institute for Health and Care Excellence (NICE) domestic violence and abuse guideline development group and led the World Health Organization (WHO) intimate partner violence guidelines.

**John Campbell**, MD, FRCGP, is professor of general practice and primary care at the University of Exeter, UK. He is a clinical trialist and health services researcher whose research focuses on measuring and monitoring the quality of primary care, particularly addressing access to care.

**Judit Konya**, MD, is an academic clinical fellow at the University of Exeter and a specialty registrar in general practice based in Cornwall. Judit has an interest in researching underserved populations.

**Concetta Perôt**, MSc, is an associate researcher with the University of Bristol and King’s College London. Concetta’s research includes peer-support for survivors of abuse, mental health needs of people using Sexual Assault Referral Centres (SARCs), and leading on the Charter for engaging abuse survivors. She is an integrative counsellor and psychotherapist, with 20 years’ social work experience, and she cofounded Survivors’ Voices.
